# Neuronal activity of the medial prefrontal cortex, nucleus accumbens, and basolateral amygdala in conditioned taste aversion and conditioned place preference induced by different doses of morphine administrations in rats

**DOI:** 10.3389/fphar.2023.1062169

**Published:** 2023-01-24

**Authors:** Chen Yin Ou, Ying Hao Yu, Chi-Wen Wu, Anna Kozłowska, Bai-Chung Shyu, Andrew Chih Wei Huang

**Affiliations:** ^1^ Department of Psychology, Fo GuangUniversity, Jiaoxi, Yilan County, Taiwan; ^2^ Department of Biotechnology and Animal Science, National ILan University, Yilan, Taiwan; ^3^ Department of Pharmacy, Keelung Hospital, Ministry of Health and Welfare, Keelung City, Taiwan; ^4^ Department of Human Physiology and Pathology, School Medicine, Collegium Medicum, University of Warmia and Mazury in Olsztyn, Olsztyn, Poland; ^5^ Institute of Biomedical Sciences, Academia Sinica, Taipei, Taiwan

**Keywords:** the paradoxical effect hypothesis of abused drugs, prelimbic cortex, infralimbic cortex, nucleus accumbens, basolateral amygdala, morphine, reward, aversion

## Abstract

To re-examine the paradoxical effect hypothesis of abused drugs, the present study concerned whether different doses of morphine disparately affect neuronal activity and associations among the subareas of the medial prefrontal cortex (mPFC: cingulate cortex 1-Cg1, prelimbic cortex-PrL, infralimbic cortex-IL), the subregions of the nucleus accumbens (NAc; both core and shell), and the basolateral amygdala (BLA) following conditioned taste aversion (CTA) and conditioned place preference (CPP). All rats were given a 0.1% saccharin solution for 15-min, and they were intraperitoneally injected with saline or 20, 30, or 40 mg/kg morphine to form the aversive CTA learning. Later, half of the rats were tested for CPP (including the CTA and then CPP tests) for 30-min. Finally, the immunohistochemical staining with c-Fos was conducted after the behavioral test. After the CTA test, c-Fos (%) in the Cg1 and PrL (but not the IL) was more in 20–40 mg/kg of the morphine groups; c-Fos (%) in the NAc core, NAc shell, and BLA was more in the 30–40 mg/kg morphine group. After the CPP test, the Cg1, PrL, IL, and BLA showed more c-Fos (%) in 20 mg/kg morphine; the NAc core showed fewer in c-Fos (%) in the 30–40 mg/kg morphine groups. The mPFC subregions (e.g., Cg1, PrL, and IL), NAc subareas (e.g., NAc core and NAc shell), and BLA were involved in the different doses of morphine injections. The correlation analysis showed that a positive correlation was observed between PrL and IL with NAc core with low doses of morphine and with NAc shell with increasing doses of morphine after the CTA test. After the CPP, an association between PrL and NAc core and NAc shell at low doses and between IL and BLA and NAc shell with increasing doses of morphine. Therefore, different neural substrates and the neural connectivity are observed following different doses of morphine and after the CTA and CPP tests. The present data extend the paradoxical effect hypothesis of abused drugs.

## 1 Introduction

Morphine abuse is characterized by psychopathological compulsion, relapse, and craving (Koob, 2000; Weiss et al., 2001). This abnormal behavior is based on the rewarding property of the abused drugs (Volkow et al., 2019). The brain reward mechanism influences misusers to crave for a drug, find access to it and consume it. Moreover, this line of research has usually used conditioned place preference (CPP) and drug self-administration to test the rewarding properties of drugs. However, fewer studies have investigated the aversion to morphine at the neural and behavioral levels to avoid morphine abuse (Solecki et al., 2009; Riley et al., 2022). Some studies have presented the aversive property of morphine in conditioned place aversion (CPA) (Wu et al., 2014) and conditioned taste aversion (CTA) tasks (Simpson and Riley, 2005). Brain glutamate and dopamine systems and the medial prefrontal cortex (mPFC) were involved in the reinforcing process (Steketee, 2003; Bishop et al., 2011; Zhao et al., 2017). The mPFC also plays an executive role in top-down processing to control reward and aversive stimuli (Riga et al., 2014). The mPFC projections to the nucleus accumbens (NAc) on dopaminergic input govern reward and aversion in cases of drug abuse and depression (Salamone and Correa, 2012). The pathway of the prelimbic cortex (PrL) projecting to the basolateral amygdala (BLA) regulated the retrieval of morphine-induced withdrawal memory in a CPA task (Song et al., 2019). Therefore, the mPFC projections to the NAc and the BLA were involved in morphine-induced conditioned rewarding and aversive learning.

**FIGURE 1:**
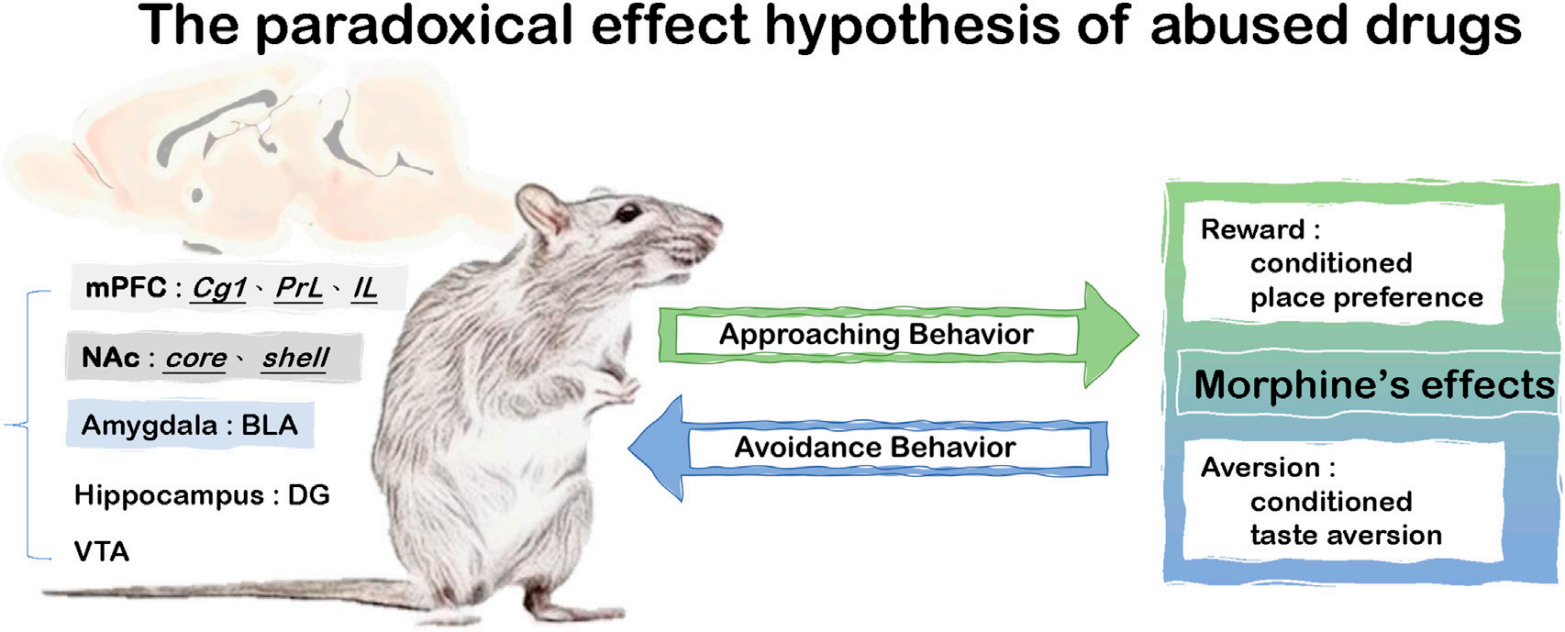
The paradoxical effect hypothesis of abused drugs.

Recently, the paradoxical effect hypothesis of abused drugs has suggested the simultaneous existence of morphine’s reward and aversion, which could affect drug misuse (Wang et al., 2021; Wu et al., 2021; Yu et al., 2021). For example, the rewarding effect of abused drugs elicits approaching behavior to access to the abused drugs and increased its probability of their abuse; in contrast, abused drugs also induce aversion which results in avoidance behaviors for the drug use (Wang et al., 2021; Wu et al., 2021; Yu et al., 2021). Furthermore, it was demonstrated many neural substrates were involved in the paradoxical effect of morphine. For example, under a 20 mg/kg dose of morphine, a lesion of the ventral tegmental area (VTA) disrupted the drug’s aversive effect in the CTA test and rewarding effect in the CPP test (Wu et al., 2021). Morphine-induced CTA conditioning could activate more c-Fos expression in the cingulate cortex 1 (Cg1), PrL, infralimbic cortex (IL), BLA, NAc, and dentate gyrus (DG) (Yu et al., 2021). Compared with the previous findings of reward learning, these data indicated that these brain areas including the Cg1, PrL, IL, BLA, NAc, and DG were also shown to be c-Fos expression after the CPP test (Yu et al., 2021). Therefore, the Cg1, PrL, IL, BLA, NAc, DG, and VTA contributed to the paradoxical effect hypothesis of morphine (see Figure 1).

However, no studies have comprehensively examined how the subareas of the mPFC (e.g., Cg1, PrL, and IL) connected to the NAc (e.g., NAc core and NAc shell) or the BLA were involved in aversion and reward under a range of low to high morphine doses. Moreover, how the Cg1, PrL, IL, NAc core, NAc shell, and BLA are involved in the morphine-induced aversion in the CTA test and reward in the CPP test after varying doses of morphine remains unknown.

This study addresses several important questions. First, can morphine induce aversion in the CTA paradigm and reward in the place conditioning paradigm? Second, whether the Cg1, PrL, IL, NAc core, NAc shell, and BLA were involved in morphine-induced aversion in the CTA test and reward in the CPP test under varying morphine dosages? Finally, are the associations of the PrL or IL with the subareas of the NAc (i.e., NAc core and NAc shell) or the BLA changed when subjected to low to high doses of morphine? Furthermore, whether the data collected could support the paradoxical effect hypothesis of abused drugs?

## 2 Materials and methods

### 2.1 Animals

For this study, 98 male Sprague Dawley rats were purchased from the BioLasco Taiwan Company, all weighing 250–350 g at the start of the experiment. All rats were raised with hardwood laboratory bedding (Beta Chip) and housed in pairs in plastic home cages (47 cm long × 26 cm wide × 21 cm high) in a colony room. The colony room was maintained at a constant temperature (approximately 23°C ± 2°C) and with a 12 h/12 h light/dark cycle (lights on 6:00–18:00). Food and water were allowed freely except during the water deprivation phase. The number of animals used was restricted, and all efforts were made to minimize the animal’s suffering. All experiments were conducted in compliance with the Animal Scientific Procedures Act of 1986. The study received approval from the Institutional Animal Care and Use Committee (IACUC) of Fo Guang University.

### 2.2 Apparatus

#### 2.2.1 Drinking device

The drinking device was composed of a 25 mL burette with 0.1 mL graduations, a white panel, and a wire mesh cage. The burette was inserted into the white panel hole and mounted in front of the cage. Animals could drink water or the 0.1% saccharin solution. The 0.1% saccharin solution consumption was analyzed as the CTA score after conditioning with morphine. The behavioral analysis for 0.1% saccharin solution consumption was provided by our previous studies (Wang et al., 2021; Yu et al., 2021).

#### 2.2.2 Place conditioning apparatus

The place conditioning apparatus had three wooden compartments and formed a T-shape. The apparatus had an intermediary shuttle compartment (33 × 16 × 16 cm high) and two distinct, approximately square compartments (49 × 42 × 35 cm high), which were defined as the conditioning compartments. Wood partitions separated the two conditioning compartments and the one shuttle compartment. The two conditioning compartments were accessible *via* the shuttle compartment. The experimenters could measure the preference behavior through a transparent acrylic glass observation wall on the front of the place conditioning apparatus. The walls of one conditioning compartment were painted with black-and-white horizontal stripes, and the compartment had a wire-grid net on the floor. Another compartment was painted white and had wood-chip bedding on the floor.

### 2.3 Behavioral procedure

**FIGURE 2:**
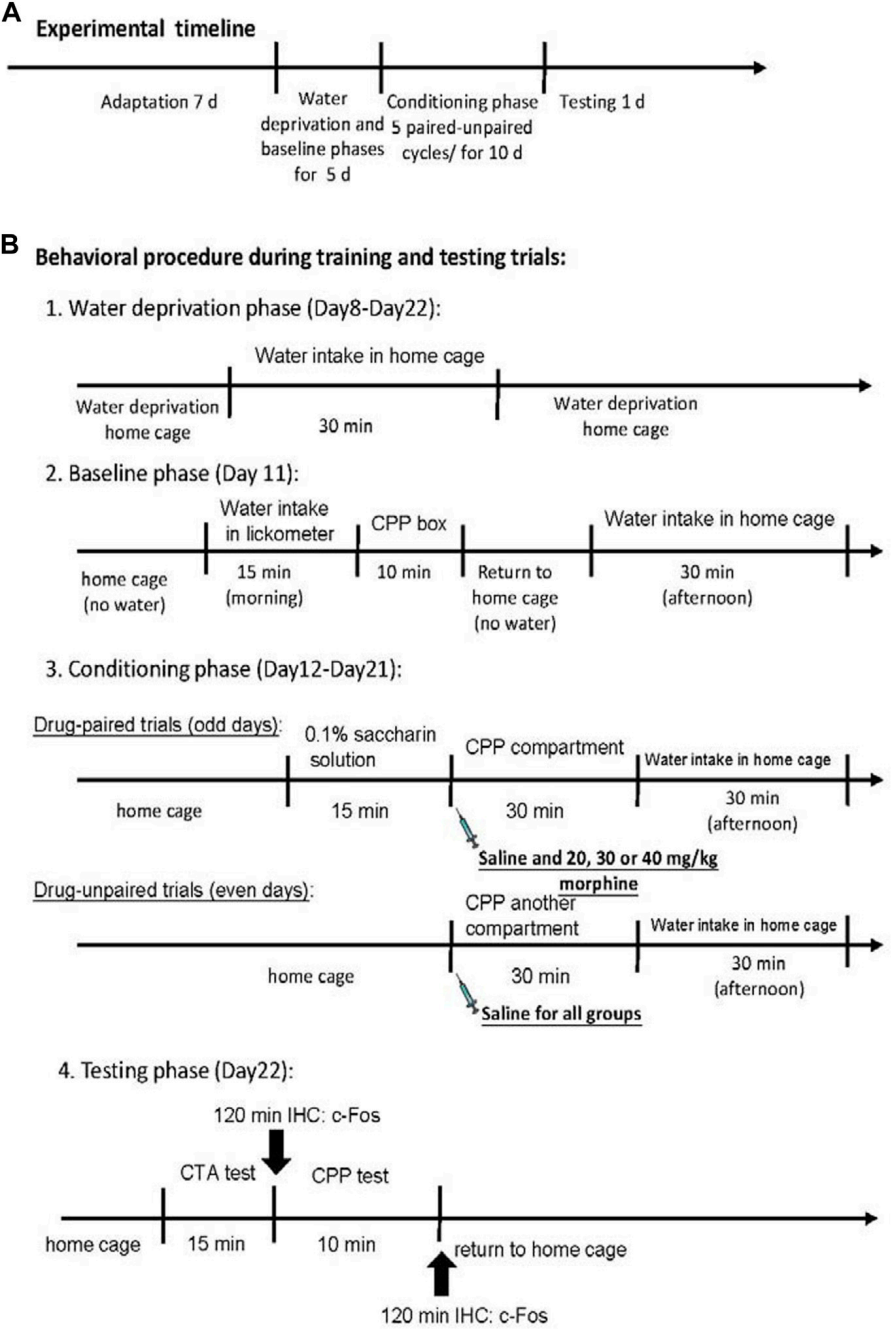
Overview of the experimental procedures. **(A)** The whole experimental timeline. **(B)** Details of behavioral procedure during training and testing trials.

At the beginning of the experiment, all rats were raised in their home cages in the colony room for 7 days during the adaptation phase. The rats were given a water-deprived regimen for 23.5 h/day during Days 8–22. During the period, all rats were allowed to drink water for 30 min in the home cage in the evening. On Day 11, the rats were trained to drink water from the lickometer for 15 min; moreover, rats were allowed to explore all compartments of the place conditioning apparatus for 10 min in the baseline phase. Later, all rats were returned to their home cage. All rats were randomly assigned to the saline, 20 mg/kg morphine, 30 mg/kg morphine, or 40 mg/kg morphine groups based on the place conditioning data. This trial phase was unbiased because the experimenter was blind to the treatments. During the paired days of the conditioning phase (Days 12, 14, 16, 18, and 20), the rats were allowed to drink a 0.1% saccharin solution for 15 min. Then, rats were given intraperitoneal saline, 20 mg/kg morphine, 30 mg/kg morphine, or 40 mg/kg morphine injections to form the CTA effect. The rats were then immediately placed in one of the place conditioning compartments of the place conditioning apparatus for 30 min to acquire the CPP effect. On the unpaired days of the conditioning phase (Days 13, 15, 17, 19, and 21), all rats stayed in their home cages without a CTA procedure. In the CPP procedure, all rats were intraperitoneally injected with the normal saline solution and put into another compartment of the place preference apparatus. During the testing phase (Day 22), the rats were tested for aversion or preference. Of the 98 rats, 49 (*n* = 12 per group except for the 40 mg/kg group were the number was *n* = 13) drank a measured amount of saccharin solution for 15 min in the CTA task. The other 49 rats (*n* = 12 per group except for the 40 mg/kg group were the number was *n* = 13) were tested in the CPP task 10 min following the 15-min CTA test. Immunohistochemical staining with c-Fos protein was performed 120 min after the final CTA or CPP test (Figure 2). Notably, some previous studies have demonstrated that morphine injections could induce different half-lives based on different methods of administering morphine in different species (Hartvig et al., 1984; Hasselstrom et al., 1990; Taylor et al., 2001; Devine et al., 2013). For example, the elimination half-life of morphine is 4.2 h for humans (Hasselstrom et al., 1990), 30–45 min for Rhesus monkeys (Hartvig et al., 1984), 1.5 h for horses (Devine et al., 2013), and 76.3 min (intravenous injections) or 93.4 min (intramuscular injections) for cats (Taylor et al., 2001). Therefore, the experimental procedure of morphine injections was staggered across Days 12, 14, 16, 18, and 20. Based on the previous data, the total drug effect of morphine was likely eliminated within 1 day. Thus, no morphine effect occurred on Days 13, 15, 17, 19, or 21. In the conditioning phase, the saccharin solution was paired with the effect of morphine injection without any other effects.

### 2.4 Immunohistochemical staining

All rats were completely unresponsive after euthanasia with an overdose of sodium pentobarbital. The rats were perfused with 0.1 M sodium phosphate-buffered saline (PBS) for the fixation procedure, followed by 4% paraformaldehyde in a 0.1 M PBS buffer. After 2 days, a post-fixation process was performed on brain tissues with a 4% paraformaldehyde solution. The brain tissues were then transferred to 30% sucrose solution in 4% paraformaldehyde for cryoprotection until the brain tissues sank to the bottom. We used a freezing and sliding microtome to cut the brain tissues into 40-μm sections through the whole brain (Paxinos and Watson, 2007).

All slices were immunohistochemically stained for c-Fos detection. The free-floating brain slices were washed for 15 min in 0.1 M PBS buffer. Later, the slices were permeabilized in 3% H_2_O_2_ for 1 h and washed for 20 min three times in 2% phosphate-buffered saline tween-20 (PBST). The brain slices were soaked in 3% normal goat serum and 1% bovine serum albumin for 1 h. After washing twice with PBST for 15 min, the slices were incubated overnight at 4°C with rabbit anti-Fos antibody (Millipore, ABE457, 1:400). The slices were washed twice for 15 min with PBST. They were incubated for 1 h with a biotinylated goat anti-rabbit secondary antibody (Vector Lab BA-1000, 1:500). The slices were washed with PBS buffer. The bound secondary antibody was applied and amplified using the ABC kit (Vector Lab ABC Kit, PK-6100). The microscope Olympus BX51 was used to assess c-Fos expression. The quantitative analysis for c-Fos expression had multiple steps. First, the reference brain area was searched using lower magnification (×4; e.g., forceps minor of the corpus callosum [fmi]). The rat brain atlas was used to confirm the target brain areas, such as the Cg1, in relation to the reference brain area (i.e., the fmi). We later enhanced the magnification to ×20 to narrow the view and took photos of the target slice. Positive expressions of c-Fos proteins in neurons were counted with ImageJ software at ×20 magnification for each slice of the entire brain (Abramoff et al., 2004). Finally, the c-Fos expression numbers were transferred into the following formula: c-Fos expression (%) = 100*(c-Fos numbers in each dose of the morphine group/averaged c-Fos expression numbers in the saline group). The c-Fos (%) was analyzed in the present study.

### 2.5 Drugs

Morphine hydrochloride was purchased from the Food and Drug Administration, Ministry of Health and Welfare, Executive Yuan (Taipei, Taiwan). Morphine hydrochloride was diluted in normal saline to a concentration of 10 mg/mL. Sodium saccharin was dissolved in distilled water to a 0.1% saccharin solution. NaCl was prepared in distilled water for a normal saline solution. Morphine was injected intraperitoneally at 2 mL/kg, 3 mL/kg, and 4 mL/kg. The doses of morphine were determined to be 20 mg/kg, 30 mg/kg, and 40 mg/kg, respectively.

### 2.6 Statistical analysis

The study used the statistical software SPSS 19.0 to analyze all data. One-way ANOVA was conducted to analyze the intake volume of the 0.1% saccharin solution. The intake volume of the saccharin solution served as the CTA index. A 4 × 5 mixed repeated-measurements ANOVA with group and session factors was used. When appropriate, *post hoc* with Fisher’s LSD tests were performed for each session. The time spent in the drug-paired chamber of the place conditioning apparatus was measured as the CPP index. A dependent *t*-test was used to analyze the time spent between the paired and unpaired compartments for all groups in the baseline and testing phases.

The intake volumes of 0.1% saccharin solution over sessions 1–6 were combined into the total intake volume. Then, the total intake volume was analyzed by a trend analysis to test the dose-response curve for different doses of morphine in CTA. Moreover, a trend analysis was used for time spent on the paired side to test the dose-response curve for different doses of morphine in CPP tests.

It was to test the correlation between the mPFC, NAc, and BLA under various doses of morphine. Spearman’s non-parametric correlation tests analyzed the c-Fos expression on the Cg1, PrL, IL, NAc core, NAc shell, and BLA. A *p*-value less than 0.05 was considered significant in all cases.

The c-Fos data (%) were analyzed separately for morphine-induced aversive learning and rewarding learning with a one-way ANOVA for all groups within the specific brain areas. When appropriate, *post hoc* with Fisher’s LSD tests were conducted. A *p*-value less than 0.05 was considered significant in all cases.

## 3 Results

### 3.1 Conditioned taste aversion and place preference by different doses of morphine

**FIGURE 3:**
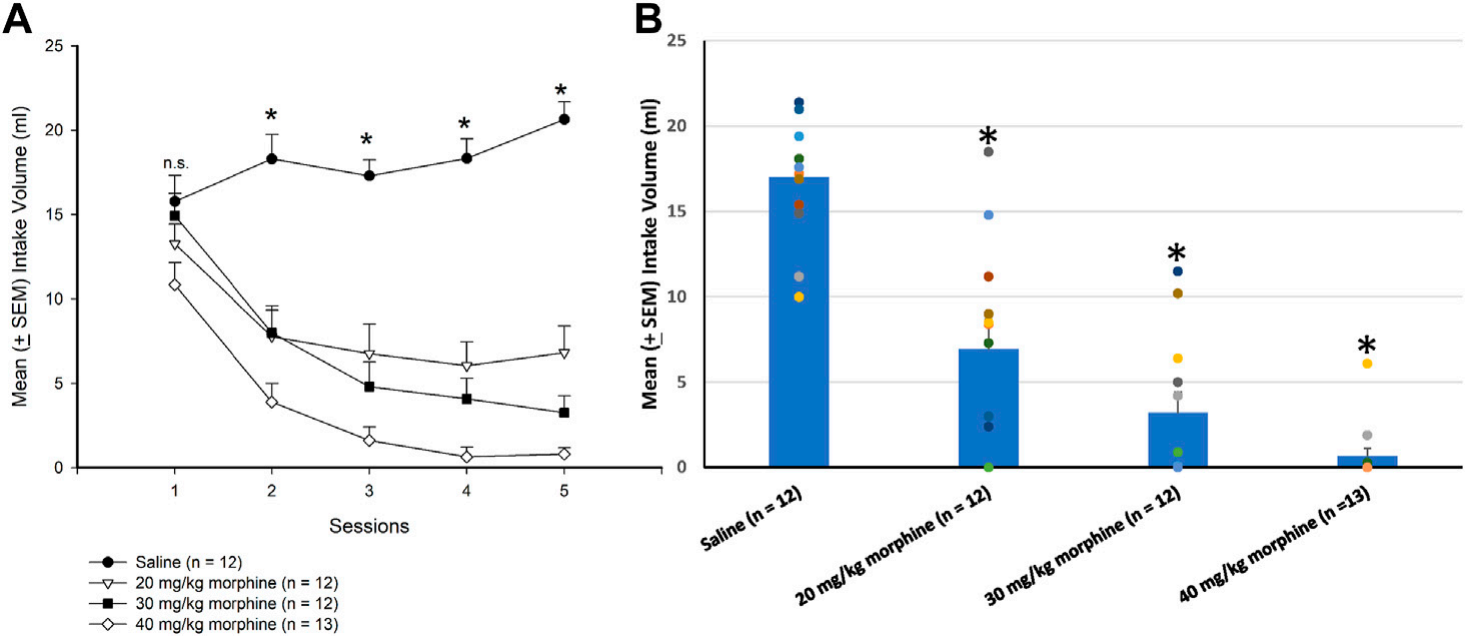
Morphine, the unconditioned stimulus agent, was conditioned with 0.1% saccharin solution to produce conditioned taste aversion (CTA). **(A)** Sessions 1–5 and **(B)** session 6 (in the testing phase) show the mean (±SEM) intake volume of 0.1% saccharin solution in rats injected with saline (*n* = 12) or with morphine in doses of 20 mg/kg (*n* = 12), 30 mg/kg (*n* = 12), and 40 mg/kg (*n* = 13). *p < 0.05 when comparing the saline groups and the various doses of morphine.

A 4 × 5 mixed two-way ANOVA was used to determine which morphine doses induced aversion in the CTA paradigm, showing that significant differences in the intake volume of saccharin solution occurred in groups [F (3, 45) = 39.93, *p* < 0.05], sessions [F (4, 180) = 37.06, *p* < 0.05], and groups × sessions [F (12, 180) = 10.59, *p* < 0.05]. Post hoc LSD tests indicated that the 20–40 mg/kg groups consumed less saccharin solution over sessions 2–5 than the saline group (*p* < 0.05; Figure 3A). Combining the intake volume of saccharin solution, a one-way ANOVA showed a significant group effect [F (3, 45) = 36.29, *p* < 0.05]. Post hoc LSD tests indicated that the 20 mg/kg, 30 mg/kg, and 40 mg/kg groups consumed significantly less saccharin solution than the saline group (*p* < 0.05; Figure 3B). Therefore, morphine administrations induced CTA learning for 20–40 mg/kg morphine groups.

**FIGURE 4:**
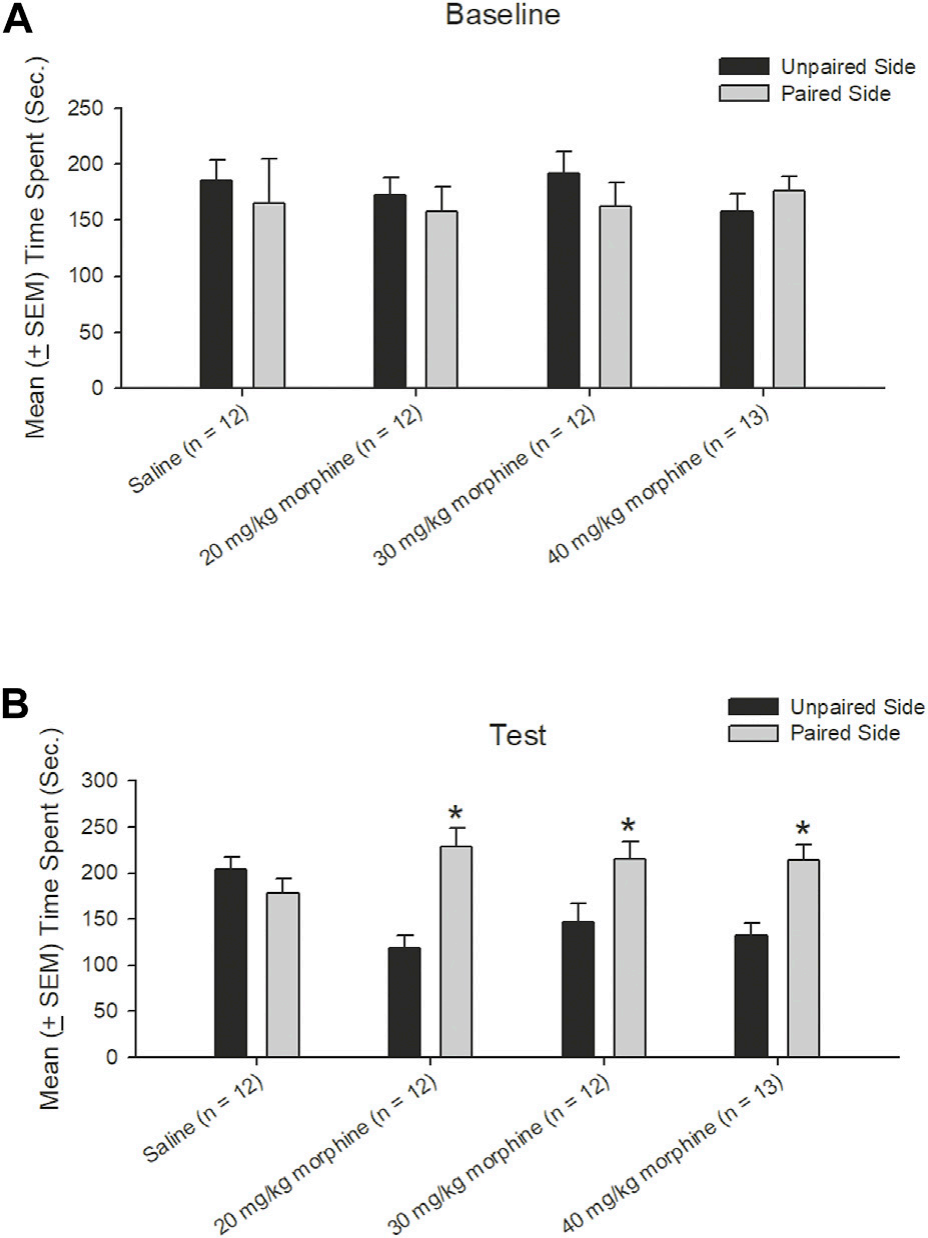
Morphine, the unconditioned stimulus agent, was paired with the contextual compartment of the conditioned place preference (CPP) task to produce the CPP effect. Mean (±SEM) time spent between unpaired and paired sides in rats injected with saline (*n* = 12) or with morphine in doses of 20 mg/kg (*n* = 12), 30 mg/kg (*n* = 12), and 40 mg/kg (*n* = 13) in **(A)** baseline and **(B)** test phases, respectively. *p < 0.05 when comparing the unpaired and paired sides in the various doses of morphine.

A dependent *t*-test was conducted to examine the rewarding effects of various doses of morphine after the CPP test. The results showed non-significant differences between unpaired and paired sides in the saline [t (11) = 0.43, *p* > 0.05], 20 mg/kg [t (11) = 0.49, *p* > 0.05], 30 mg/kg [t (11) = 0.89, *p* > 0.05], and 40 mg/kg [t (12) = −0.70, *p* > 0.05] groups in the baseline phase (Figure 4A). In the test phase, significant increases were observed in the time spent in the drug-paired side compared to the unpaired side in the 20 mg/kg [t (11) = −4.47, *p* < 0.05], 30 mg/kg [t (11) = −2.19, *p* = 0.05], and 40 mg/kg [t (12) = −3.46, *p* < 0.05] morphine groups; however, no significant difference existed in time spent between the paired and unpaired sides of the saline group [t (11) = 0.97, *p* > 0.05; Figure 4B]. Therefore, morphine induced conditioned place preference for 20–40 mg/kg morphine groups.

**FIGURE 5:**
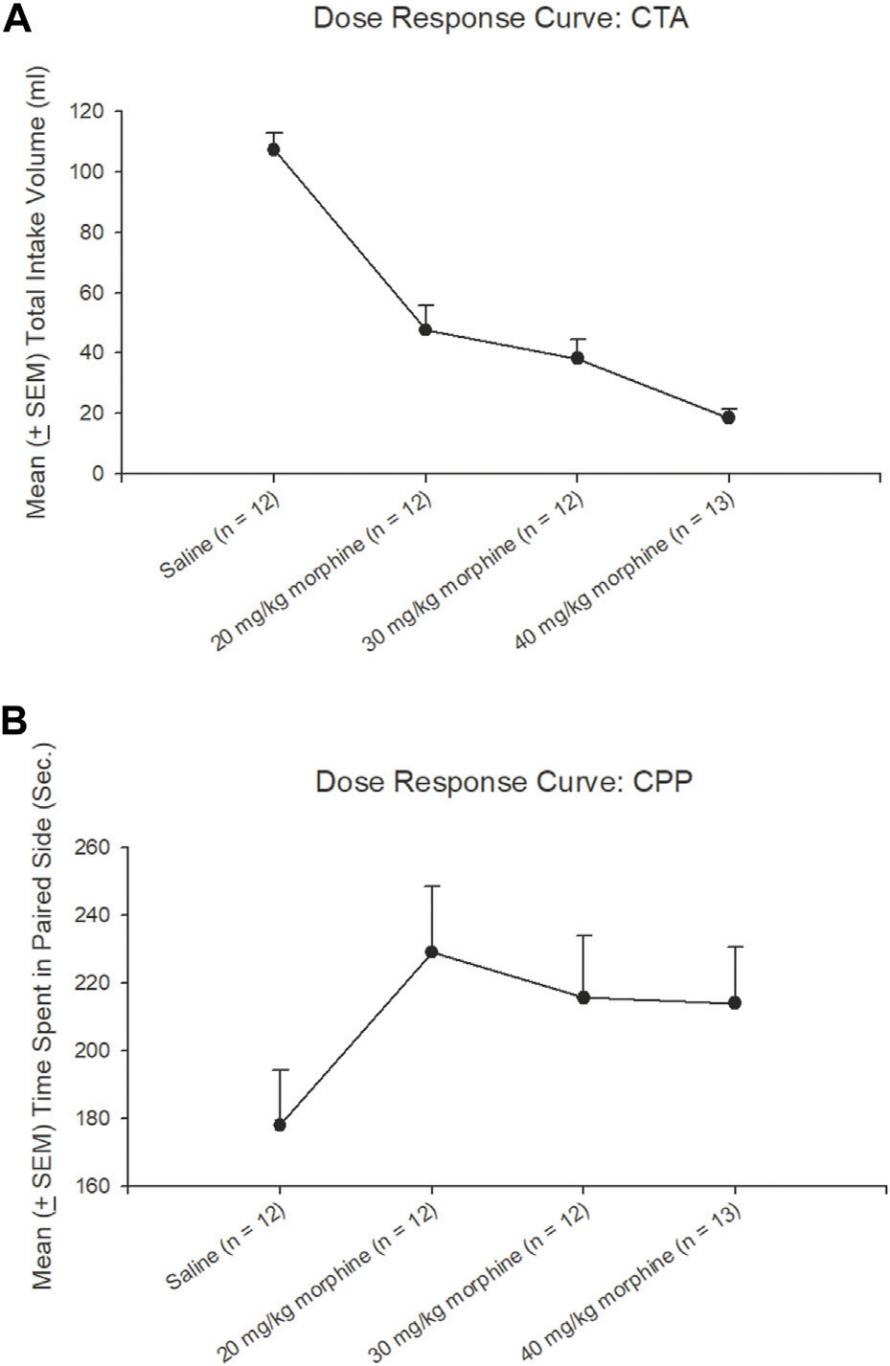
Dose-response curve tests under different doses of morphine injections or saline (*n* = 12), 20 mg/kg (*n* = 12), 30 mg/kg (*n* = 12), and 40 mg/kg (*n* = 13) in **(A)** conditioned taste aversion (CTA) and **(B)** conditioned place preference (CPP) during test phases, respectively.

The dose-response curve for different doses of morphine administrations after the CTA test, a trend analysis showed that linear [F (1, 11) = 150.73, *p* < 0.05], quadratic [F (1, 11) = 9.23, *p* < 0.05], and cubic [F (1, 11) = 3.60, *p* > 0.05], indicating the CTA’s dose-response cure was a quadratic U-shape. The total intake volume of 20 mg/kg, 30 mg/kg, and 40 mg/kg morphine groups were significantly decreased compared to the saline group (*p* < 0.05); however, the 30 mg/kg and 40 mg/kg doses appeared to have a slower trend for the decline of the CTA effect. The results showed the dose-response curve for the CTA tests after different doses of morphine injections (Figure 5A).

Concerning the CPP tests, a trend analysis showed that linear [F (1, 11) = 1.16, *p* > 0.05], quadratic [F (1, 11) = 4.90, *p* < 0.05], and cubic [F (1, 11) = 0.94, *p* > 0.05], indicating the CPP’s dose-response curve was linear increasing from the saline to 20 mg/kg morphine group and still remained at a high level with the remaining doses. The time spent on the paired side of 20–40 mg/kg morphine groups was progressively increased compared to the saline group. Therefore, the different doses of morphine injections showed a dose-response curve for CPP tests (Figure 5B).

### 3.2 Neural substrates were involved in morphine’s reward and aversion: c-Fos data

**FIGURE 6:**
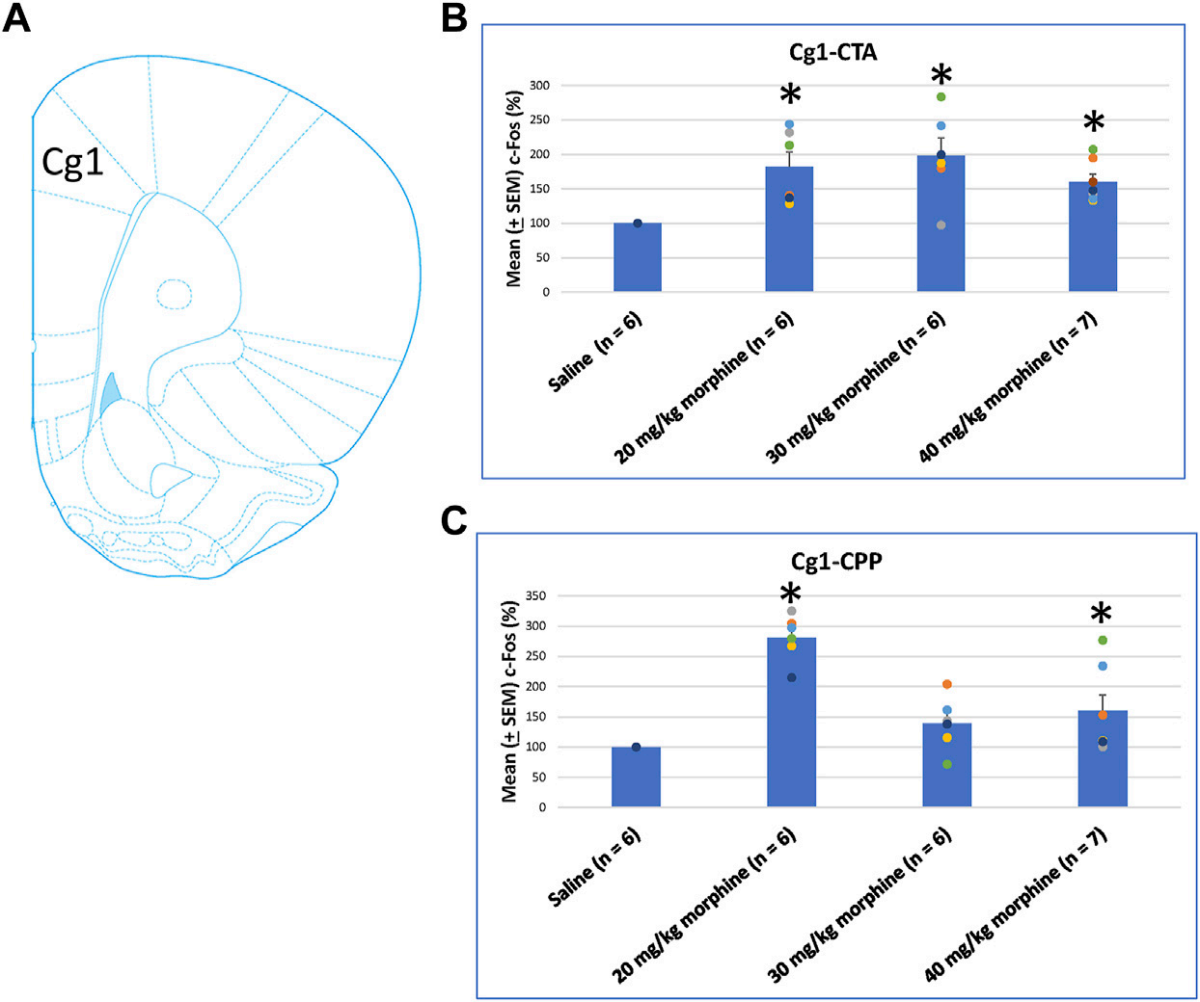
**(A)** Representative image of c-Fos immunoreactivity in cingulated cortex area 1 (Cg1). Mean (±SEM) c-Fos (%) in Cg1 following **(B)** CTA and **(C)** CPP tests. *p < 0.05 when comparing the saline for CTA and CPP (both, *n* = 6) or with morphine in doses of 20 mg/kg for CTA and CPP (both, *n* = 6), 30 mg/kg for CTA and CPP (both, *n* = 6), and 40 mg/kg for CTA (*n* = 6) and CPP (*n* = 7).

After specific doses of morphine-induced an aversive effect in the CTA paradigm and a rewarding effect in the place preference paradigm, neural substrates were examined to determine their involvement in both tests. One-way ANOVA showed a significant effect between groups [F (3, 21) = 6.00, *p* < 0.05]. Post hoc tests with Fisher’s LSD indicated that the 20–40 mg/kg groups showed significantly increased c-Fos (%) in the C1g after CTA tests compared to the saline group (*p* < 0.05; Figure 6B). Moreover, one-way ANOVA indicated significant group differences after CPP tests [F (3, 21) = 17.03, *p* < 0.05]. A *post hoc* test with Fisher’s LSD indicated that c-Fos (%) also significantly increased in the Cg1 in the 20 mg/kg and 40 mg/kg morphine groups (*p* < 0.05) but displayed non-significant differences in the 30 mg/kg group (*p* > 0.05; Figure 6C; Supplementary Figure S1). Therefore, c-Fos (%) was more in the 20–40 mg/kg morphine groups compared with the saline group following CTA tests; moreover, c-Fos (%) was more in the 20 mg/kg and 40 mg/kg morphine groups compared with the saline group after CPP tests.

**FIGURE 7:**
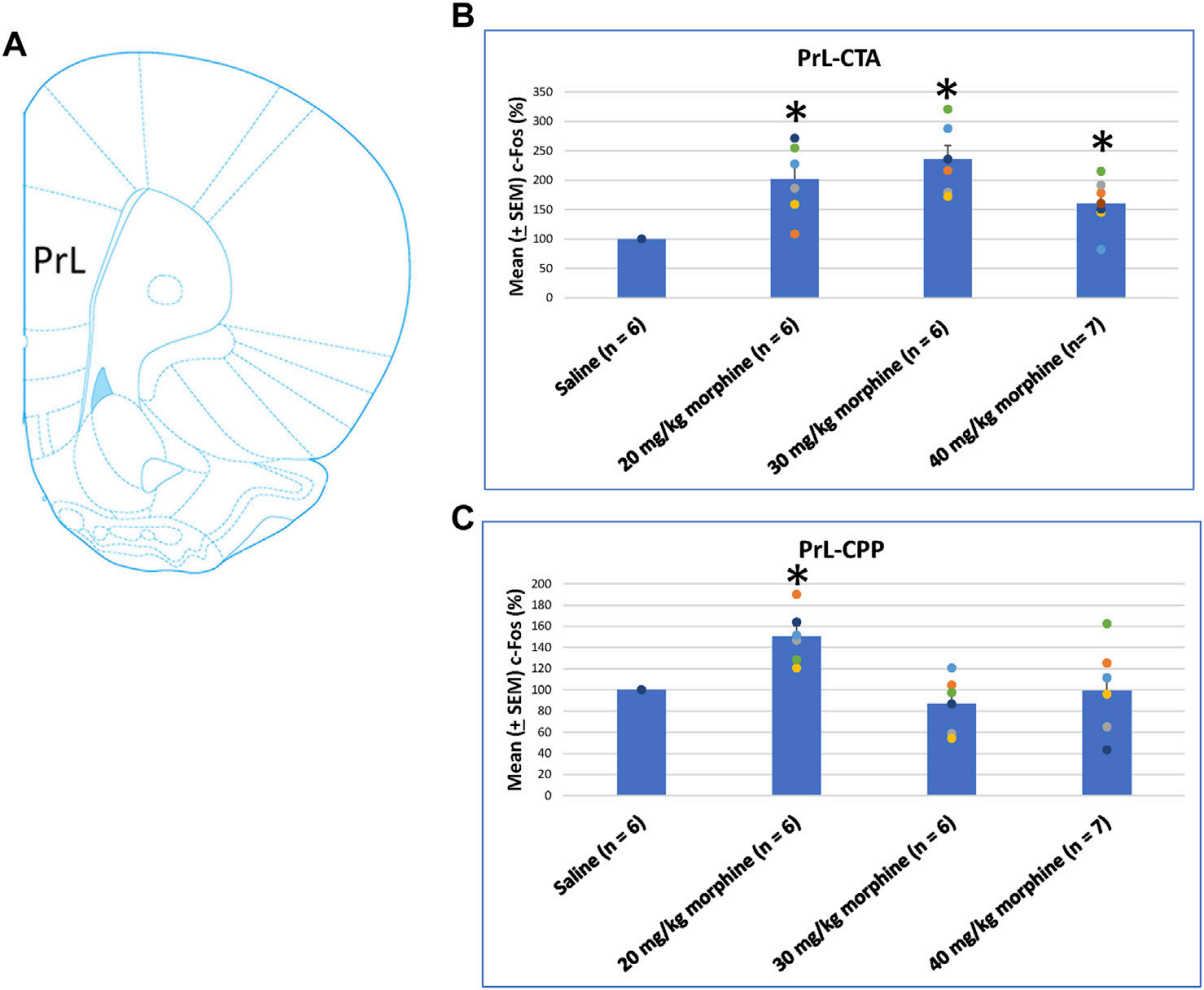
**(A)** Representative image of c-Fos immunoreactivity in the prelimbic cortex (PrL). Mean (±SEM) c-Fos (%) in PrL in the following **(B)** CTA and **(C)** CPP tests. *p < 0.05 when comparing the saline for CTA and CPP (both, *n* = 6) or with morphine in doses of 20 mg/kg for CTA and CPP (both, *n* = 6), 30 mg/kg for CTA and CPP (both, *n* = 6), and 40 mg/kg for CTA (*n* = 6) and CPP (*n* = 7).

When assessing the c-Fos (%) in PrL following morphine-induced CTA and CPP effects, the results of CTA tests showed significant group differences [F (3, 21) = 9.07, *p* < 0.05]. Post hoc analysis with Fisher’s LSD tests showed significant increases in the 20–40 mg/kg morphine groups (*p* < 0.05; Figure 7B). In CPP tests for c-Fos (%), a significant difference occurred at the group level [F (3, 21) = 6.34, *p* < 0.05]. The *post hoc* Fisher’s LSD tests indicated that c-Fos (%) in the 20 mg/kg group significantly increased more than in the saline group (*p* < 0.05). However, the 30–40 mg/kg morphine groups did not show significant differences in c-Fos (%) (*p* > 0.05). Therefore, the PrL appeared to be involved in the morphine-induced rewarding effect in CPP tests under the 20 mg/kg morphine dose (Figure 7C; Supplementary Figure S2). Therefore, c-Fos (%) in the PrL was more in the 20–40 mg/kg morphine groups than in the saline group after CTA tests. However, c-Fos (%) in the PrL was more in the 20 mg/kg morphine group after CPP tests.

**FIGURE 8:**
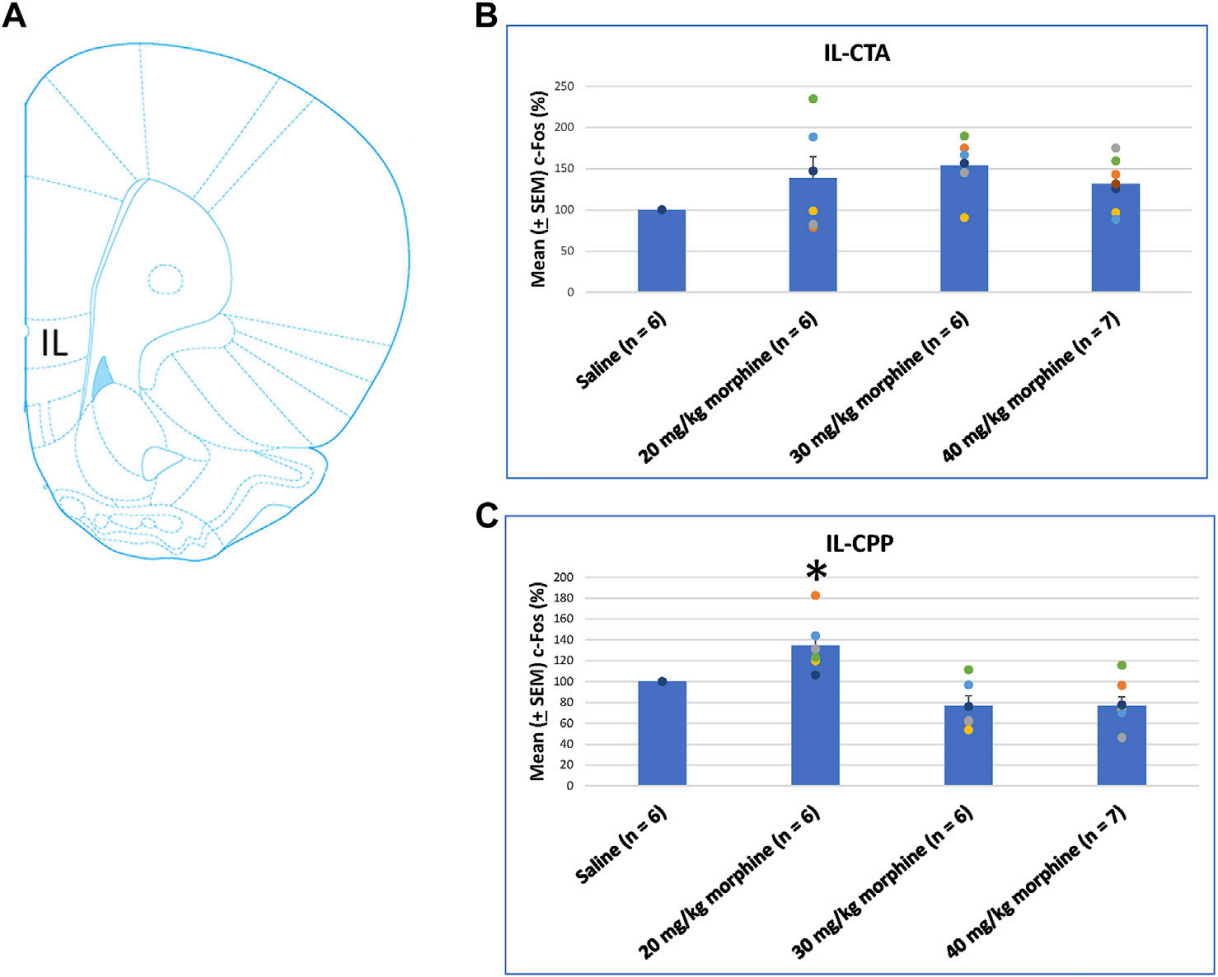
**(A)** Representative image of c-Fos immunoreactivity in the infralimbic cortex (IL). Mean (±SEM) c-Fos (%) in the IL in the following **(B)** CTA and **(C)** CPP tests. Non-significant differences occurred when comparing the saline for CTA and CPP (both, *n* = 6) or with morphine in doses of 20 mg/kg for CTA and CPP (both, *n* = 6), 30 mg/kg for CTA and CPP (both, *n* = 6), and 40 mg/kg for CTA (*n* = 6) and CPP (*n* = 7).

Concerning the effects of different doses of morphine in the CTA and CPP tasks on c-Fos (%), the IL showed non-significant differences between all morphine dose levels [F (3, 21) = 2.02, *p* > 0.05] in CTA (Figure 8B). However, significant differences occurred in the IL [F (3, 21) = 10.34, *p* < 0.05] in CPP (Figure 8C). Post hoc Fisher’s LSD tests indicated a significant increase in c-Fos (%) for the 20 mg/kg morphine group (*p* < 0.05). Non-significant differences occurred in the 30 and 40 mg/kg groups compared to the saline group (*p* > 0.05; Figure 8C; Supplementary Figure S3). Therefore, c-Fos expression in the IL was more in the 20 mg/kg morphine group compared with the saline group after the CPP test.

**FIGURE 9:**
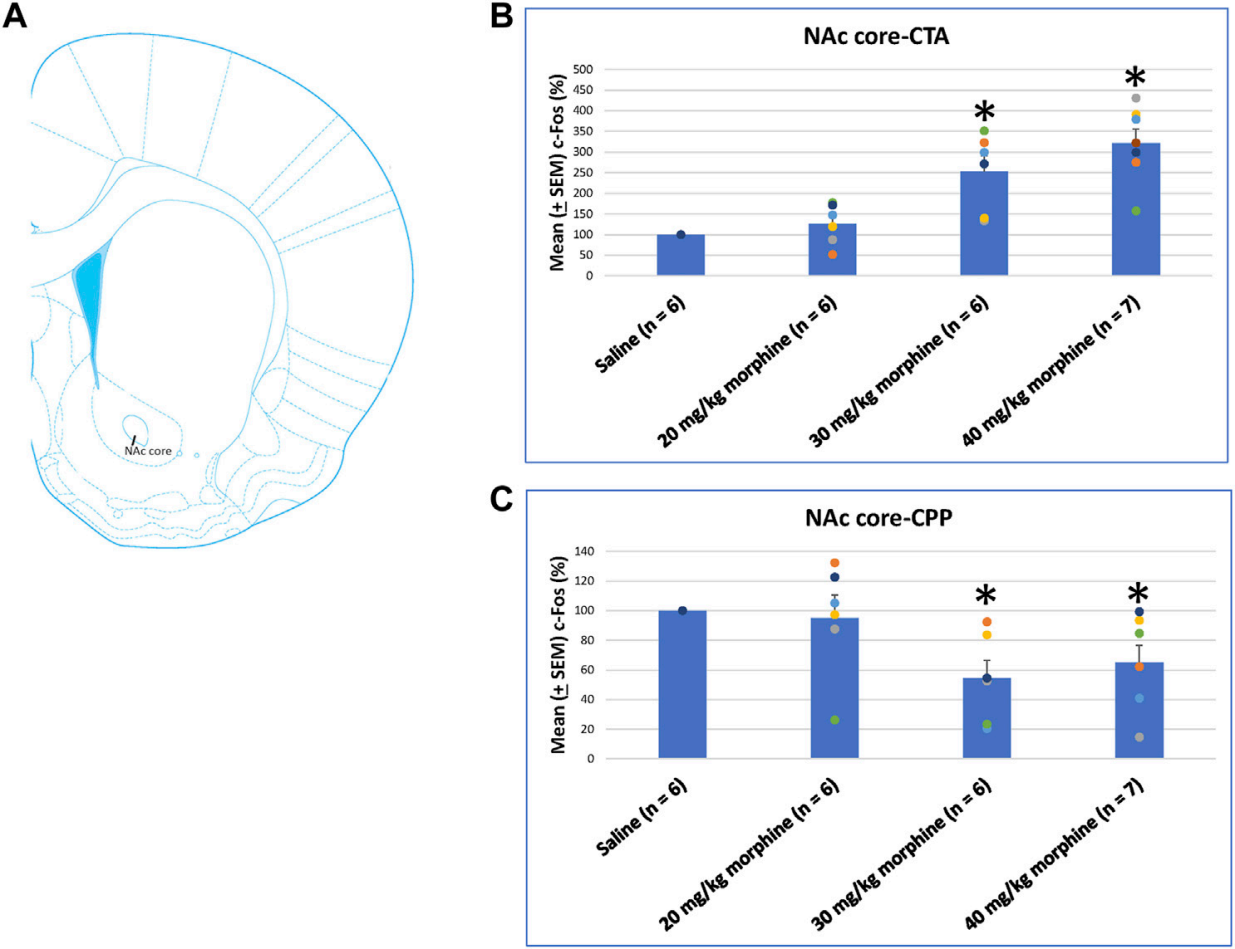
**(A)** Representative image of c-Fos immunoreactivity in the nucleus accumbens core (NAc core). Mean (±SEM) c-Fos (%) in the NAc core following **(B)** CTA and **(C)** CPP tests. *p < 0.05 when comparing the saline for CTA and CPP (both, *n* = 6) or with morphine in doses of 20 mg/kg for CTA and CPP (both, *n* = 6), 30 mg/kg for CTA and CPP (both, *n* = 6), and 40 mg/kg for CTA (*n* = 6) and CPP (*n* = 7).

The NAc core showed significant differences in c-Fos (%) after CTA tests [F (3, 21) = 14.11, *p* < 0.05]. Post hoc Fisher’s LSD tests also indicated that the 30 mg/kg and 40 mg/kg groups showed significantly increased c-Fos (%) after CTA tests (*p* < 0.05; Figure 9B). In CPP tests, the group differences were significant [F (3, 21) = 3.78, *p* < 0.05; Figure 9C; Supplementary Figure S4]. Post hoc Fisher’s LSD tests indicated that c-Fos (%) of the 30 mg/kg and 40 mg/kg morphine groups was significantly decreased compared to the saline group (*p* < 0.05). Therefore, c-Fos (%) in the NAc core was more in the 30–40 mg/kg morphine groups compared with the saline group after CTA tests; however, c-Fos (%) in the NAc core was fewer in the 30–40 mg/kg morphine groups after CPP tests.

**FIGURE 10:**
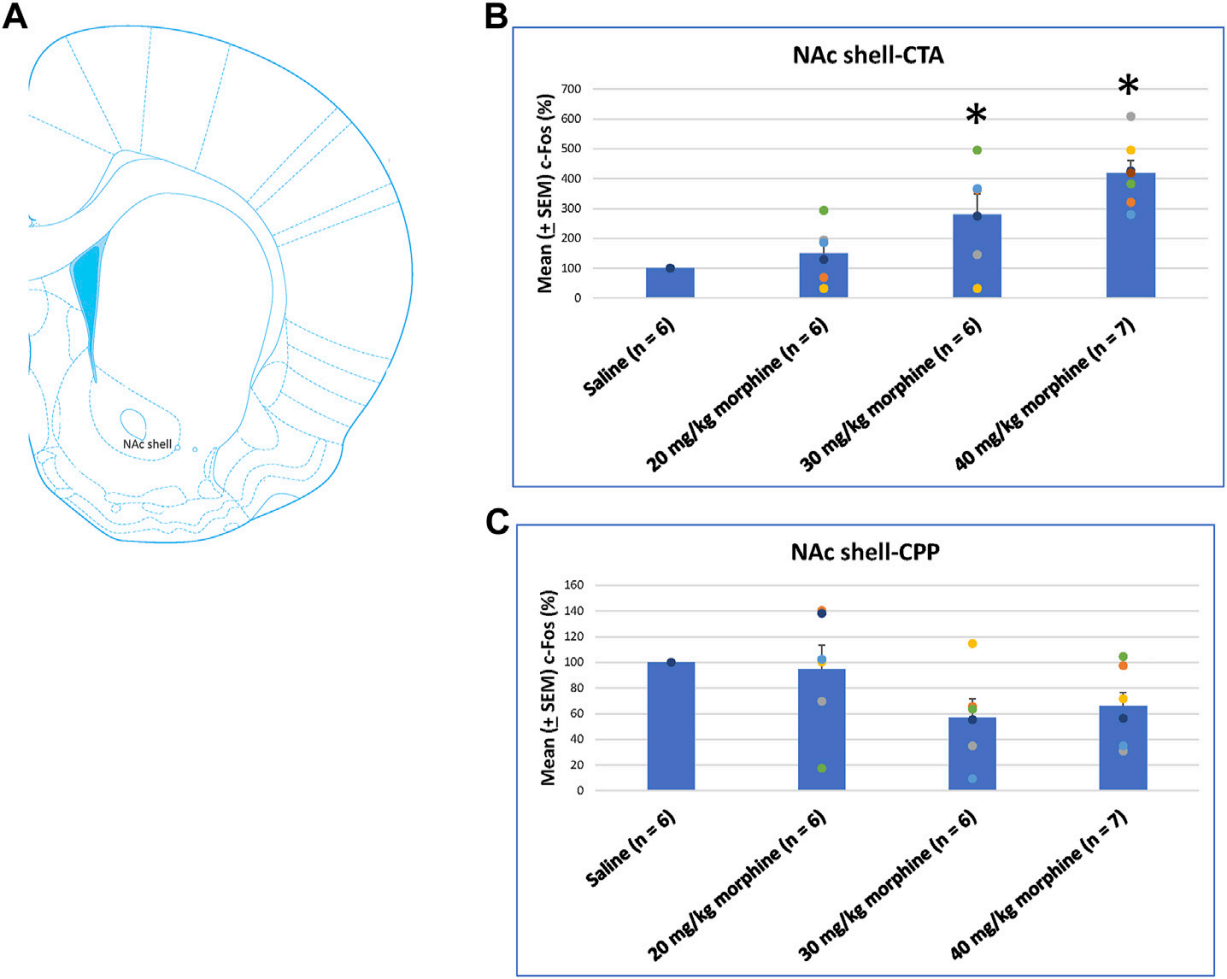
**(A)** Representative image of c-Fos immunoreactivity in the nucleus accumbens shell (NAc shell). Mean (±SEM) c-Fos (%) in the NAc shell following **(B)** CTA and **(C)** CPP tests. *p < 0.05 when comparing the saline for CTA and CPP (both, *n* = 6) or with morphine in doses of 20 mg/kg for CTA and CPP (both, *n* = 6), 30 mg/kg for CTA and CPP (both, *n* = 6), and 40 mg/kg for CTA (*n* = 6) and CPP (*n* = 7).

A one-way ANOVA was used to measure c-Fos (%) in the NAc shell following the induction of the CTA and CPP learning with different doses of morphine. The results showed significant differences among the groups after CTA tests [F (3, 21) = 10.83, *p* < 0.05]. Post hoc Fisher’s LSD tests indicated that the 30–40 mg/kg groups significantly increased c-Fos (%) compared to the saline group for the CTA test (*p* < 0.05; Figure 10B). In the CPP tests, the group differences were non-significant for all groups for the CPP test [F (3, 21) = 2.65, *p* > 0.05; Figure 10C; Supplementary Figure S5]. Therefore, c-Fos (%) in the NAc shell was more in the 30–40 mg/kg morphine groups compared with the saline group after CTA tests.

**FIGURE 11:**
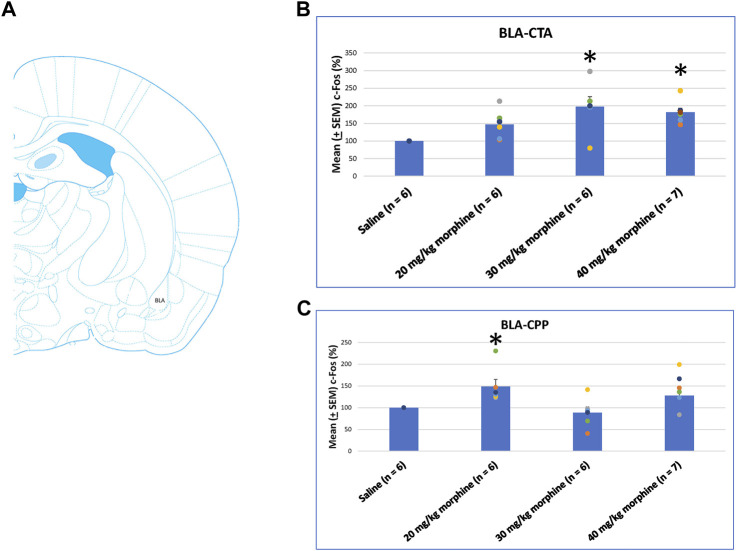
**(A)** Representative image of c-Fos immunoreactivity in the basolateral amygdala (BLA). Mean (±SEM) c-Fos (%) in the BLA in the following **(B)** CTA and **(C)** CPP tests. *p < 0.05 when comparing the saline for CTA and CPP (both, *n* = 6) or with morphine in doses of 20 mg/kg for CTA and CPP (both, *n* = 6), 30 mg/kg for CTA and CPP (both, *n* = 6), and 40 mg/kg for CTA (*n* = 6) and CPP (*n* = 7).

A one-way ANOVA was conducted separately to test c-Fos (%) in the BLA after the induction of the CTA and CPP conditioned learning with different doses of morphine. The results of the CTA test showed that the groups had significant differences in c-Fos (%) [F (3, 21) = 6.28, *p* < 0.05]. A *post hoc* Fisher’s LSD test indicated that the 30 mg/kg and 40 mg/kg groups appeared to increase c-Fos (%) significantly compared to the saline group (*p* < 0.05; Figure 11B). For the CPP test, the group differences in c-Fos (%) were significant for all groups [F (3, 21) = 3.13, *p* < 0.05; Figure 11C; Supplementary Figure S6]. Therefore, c-Fos (%) in the BLA was more in the 30–40 mg/kg morphine groups compared with the saline group after CTA tests. Moreover, c-Fos (%) in the BLA was more in the 20 mg/kg morphine group compared with the saline group after CPP tests.

### 3.3 c-Fos expression numbers and correlation analysis among selected brain areas following morphine reward and aversion

**FIGURE 12:**
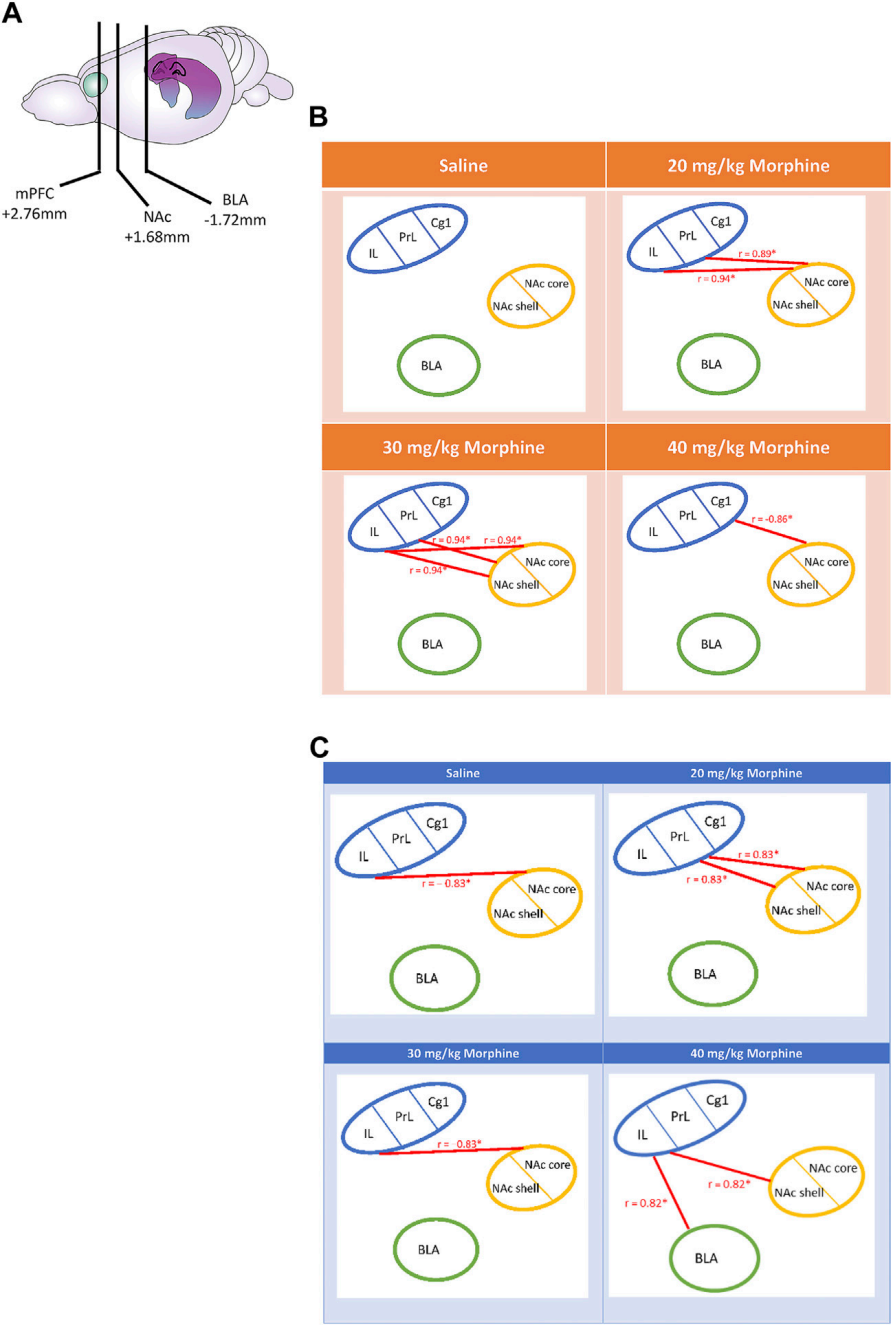
**(A)** The carton depicts the selected brain sites and their locations, including the mPFC, NAc, and BLA. After various doses of morphine-induced **(B)** CTA and **(C)** CPP, the correlation tests were performed on the Cg1, PrL, IL, NAc core, NAc shell, and BLA. *p < 0.05 was a significant difference.

Following CTA tests, c-Fos data for the saline group showed a non-significant correlation in the relationship between the mPFC (Cg1, PrL, and IL), NAc core, NAc shell, and BLA brain areas (*p* > 0.05). In the 20 mg/kg group, the PrL (r = 0.89, *p* < 0.05) and IL (r = 0.94, *p* < 0.05) were positively correlated with the NAc core. In the 30 mg/kg group, the PrL (r = 0.94, *p* < 0.05) and IL (r = 0.94, *p* < 0.05) were positively correlated with the NAc shell; moreover, the IL was positively correlated with the NAc core (r = 0.94, *p* < 0.05). In the 40 mg/kg group, the Cg1 was negatively correlated with the NAc core (r = −0.86, *p* < 0.05; Figure 12B). In summary, the connectivity from the PrL and IL to NAc core moved to the connectivity from the PrL and IL to NAc shell after CTA tests.

Regarding the mPFC’s Cg1, PrL, and IL, NAc core, NAc shell, and BLA after the CPP tests, the c-Fos data of the saline group showed that the IL was negatively correlated with the NAc core (r = −0.83, *p* < 0.05). In the 20 mg/kg group, the PrL was positively correlated with the NAc core (r = 0.83, *p* < 0.05) and the NAc shell (r = 0.83, *p* < 0.05). In the 30 mg/kg group, the IL was negatively correlated with the NAc core (r = −0.83, *p* < 0.05). In the 40 mg/kg group, the IL was positively correlated with the BLA (r = 0.82, *p* < 0.05) and the NAc shell (r = 0.82, *p* < 0.05; Figure 12C). Therefore, the correlation analysis seemingly revealed that the connections from the PrL to the NAc core and NAc shell moved to the connections of the IL to the NAc shell and BLA after CPP tests.

In summary, the correlation data after CTA tests showed that the positive correlation of the PrL and IL with the NAc core in the 20 mg/kg changed to a positive correlation with the NAc shell in the 30 mg/kg group.

With doses of morphine increasing from low to high (20 mg/kg to 40 mg/kg) after CPP tests, the association changed from the PrL projecting to the NAc core and the NAc shell to an association of the IL with the NAc shell and the BLA. In contrast, the correlation data in the 20 mg/kg group showed that the PrL was positively connected with the NAc shell and the NAc core. For the highest dose (40 mg/kg morphine), the association changed from the PrL to the IL, which was positively connected to the BLA and NAc shell. Notably, the 30 mg/kg and the saline groups showed a negative correlation between the IL and the NAc core.

## 4 Discussion

Different doses of morphine were conditioned with saccharin solution and contextual cues to simultaneously produce CTA and CPP. A dose of morphine equal to or higher than 20 mg/kg could induce the aversive CTA and the rewarding CPP effects on behavioral levels.

We analyzed the correlation analysis among the mPFC subareas, the BLA, and the subareas of the NAc following the aversive CTA test and the rewarding CPP test. Following the morphine-induced CTA tests, the correlation data showed that the 20–30 mg/kg morphine administrations showed a positive connection change from the PrL and IL connection with the NAC core to the NAc shell. Furthermore, the Cg1 was negatively connected to the NAc core. After the morphine-induced CPP tests, the doses of morphine from low to high (20 mg/kg to 40 mg/kg) showed that the connection changed from the PrL projecting to the NAc shell and the NAc core to the IL projecting to the NAc shell and BLA in the morphine-induced CPP.

We found that the specific brain areas (including the PrL, NAc core, and NAc shell) expressed significantly different levels of c-Fos expression in the saline group. Therefore, we transferred the c-Fos expression numbers into c-Fos (%) for each dose of the morphine group. After CTA tests, the data of c-Fos (%) showed that the Cg1and PrL showed high expression levels in the 20–40 mg/kg morphine groups; the NAc core, NAc shell, and BLA expressed high c-Fos levels in the 30 mg/kg and 40 mg/kg morphine groups. However, the IL showed non-significant differences in c-Fos (%) for all doses of morphine compared with the saline group (Table 1).

**TABLE 1:** Summary of c-Fos (%) in specific brain areas for various doses of morphine injections after morphine-induced conditioned taste aversion (CTA).

Morphine doses (mg/kg)				
	0	20	30	40
Cg1	—	↑	↑	↑
PrL	—	↑	↑	↑
IL	—	—	—	—
NAc core	—	—	↑	↑
NAc shell	—	—	↑	↑
BLA	—	—	↑	↑

—, non-significant difference; **↑**, significant increases; Cg1, cingulated cortex area 1; PrL, prelimbic cortex; IL, infralimbic cortex; NAc core, nucleus-accumbens core; NAc shell, nucleus accumbens shell; BLA, basolateral amygdala.

After CPP tests, the c-Fos (%) expression in the Cg1 significantly increased in the 20 mg/kg and 40 mg/kg morphine groups compared with the saline group. The c-Fos (%) of the PrL, IL, and BLA appeared more in the 20 mg/kg morphine group. However, the NAc core showed significant decreases in c-Fos (%) for the 30 mg/kg and 40 mg/kg morphine groups. The other brain area (e.g., the NAc shell) did not show any significant differences in c-Fos (%) in any doses of morphine groups (Table 2).

**TABLE 2:** Summary of c-Fos (%) in specific brain areas for various doses of morphine injections after morphine-induced conditioned place preference (CPP).

Morphine doses (mg/kg)				
	0	20	30	40
Cg1	—	↑	—	↑
PrL	—	↑	—	—
IL	—	↑	—	—
NAc core	—	—	↓	↓
NAc shell	—	—	—	—
BLA	—	↑	—	—

—, non-significant difference; ↑, significant increases; ↓, significant decreases; Cg1, cingulate cortex area 1; PrL, prelimbic cortex; IL, infralimbic cortex; NAc core, nucleus accumbens core; NAc shell, nucleus accumbens shell; BLA, basolateral amygdala.

### 4.1 Neural correlation among the mPFC, NAc, and BLA in rats expressed CTA and CPP

Previously, no research has comprehensively examined the connections between the mPFC, NAc, and BLA under various doses of morphine. Some studies have suggested that the neural projections from the mPFC to either the NAc or the BLA were involved in the rewarding and aversive effects of morphine (Ventura et al., 2005; Bassareo et al., 2007; Zhao et al., 2017; Song et al., 2019; Huang et al., 2020). In a previous study, when the inhibitory eNpHR virus was microinjected into the BLA and the virus infected the PrL, the optical stimulation in the PrL resulted in disruptions of morphine-induced withdrawal effect during the CPA task. This indicated that the PrL projecting to the BLA mediated the retrieval of morphine-induced aversive withdrawal behaviors (Song et al., 2019). Following chronic morphine treatments, morphine-induced aversive withdrawal memory with cue retrieval has mediated by the projections from the mPFC to the BLA *via* miR-105-modulation of D1 receptors (Zhao et al., 2017). Our previous study found that the excitation of PrL neurons with low concentrations of NMDA microinjected in this brain area disrupted morphine-induced CTA and decreased c-Fos expression in the BLA. The NMDA lesions created with high-concentration of NMDA enhanced morphine-induced CTA learning and increased BLA neuron activity (Huang et al., 2020). Additionally, activating the BLA neurons with low-concentration of NMDA enhanced morphine-induced CTA learning and inhibited PrL neuron activity. In contrast, BLA lesions by high concentrations of NMDA microinjected in this brain area interfered with morphine-induced CTA and increased c-Fos expression in the PrL. This suggested that the PrL and BLA had balancing roles in morphine-induced CTA (Huang et al., 2020). A microdialysis study showed that the cue-related drug stimulated dopamine releases in the NAc shell and mPFC; however, this cue did not affect dopamine transmission in the NAc core after morphine conditioning. The NAc shell and mPFC, but not the NAc core, regulate reward learning under morphine (Bassareo et al., 2007). Specific lesions to the mPFC noradrenergic transmission projecting to the NAc interfered with morphine-induced CPP conditioning and reinstatement and the downstream brain area, the NAc (Ventura et al., 2005).

In conclusion, the mPFC appears to be connected to the BLA and the NAc in governing morphine-induced reward and aversion learning. Importantly, the present data were consistent with the evidence above. The study also showed that the associations of the PrL and IL changed from the NAc core to the NAc shell in morphine-induced aversive CTA learning under morphine doses varying from low to high. In contrast, with increasing doses of morphine, the PrL was positively correlated with the NAc core and NAc shell; then, the positive association changed to that of the IL to the NAc shell and the BLA in a morphine-induced rewarding CPP learning. Therefore, the data showed that changing morphine’s doses were from low to high after the morphine-induced aversive learning, and the functional activity of the mPFC (i.e., PrL and IL) was shifted from the NAc core to the NAc shell under the morphine’s CTA learning. Following the morphine-induced CPP learning, changing morphine’s doses were from low to high and the functional connectivity of the mPFC was shifted from the PrL to the NAc core and NAc shell and from the IL to the BLA and NAc shell under the morphine’s CPP learning.

The results indicated that the mPFC, NAc, and BLA played an essential role to connect with each other for morphine’s different doses-induced aversion in the CTA task and reward in the CPP task. However, the PrL and IL of the mPFC (but not Cg1) had a positively functional connectivity with the NAc core with a low dose of morphine and with the NAc shell with high morphine doses under morphine’s aversive effect. It indicated that the subarea of the NAc has multiple functions for the aversion at the different doses of morphine, but the PrL and IL maintained the constant role to be involved in the aversive effect in low and high doses of morphine.

In contrast, under the low dose of morphine-induced CPP learning, the PrL was positively associated with the NAc core and NAc shell; however, in the high dose of morphine-induced CPP learning, the functional connectivity was shifted from the IL to the NAc shell and BLA. The changes in morphine’s rewarding effect are different than that in morphine’s aversive effect. In contrast to the previous findings in the paradoxical effect hypothesis of abused drugs, this study further examined, under various doses, morphine-induced reward- and aversion-conditioned learning. The changes in the connectivity from the mPFC subareas of PrL and IL to the subregions of the NAc (i.e., the NAc core and NAc shell) or the BLA should receive attention. The present findings need to be considered for developing novel interventions to morphine addiction. Further studies should investigate whether these connection changes indicate a need for alterations to clinical treatments.

### 4.2 Do the mPFC, NAc, and BLA contribute to morphine’s reward and aversion?

A growing body of evidence shows that the mPFC and its subareas, including the PrL, IL, and Cg1, are involved in morphine’s reward and aversion (Giacchino and Henriksen, 1998; Hao et al., 2007; Hao et al., 2008; Shahidani et al., 2012; Tan et al., 2014; Aboutalebi et al., 2018; Jamali et al., 2021). For example, lesions in the mPFC disrupted the morphine-induced rewarding effect in conditioning and reinstatement (Hao et al., 2008), behavioral sensitization in the development phase (Hao et al., 2007), and dopamine transmission in the VTA (Shahidani et al., 2012). Microinjections of orexin-1 receptor antagonist into the mPFC attenuated the morphine-induced CPP (Jamali et al., 2021). An NMDA receptor blockade of the PrL activated the dopamine neurons in the sub-cortical VTA, indicating the PrL NMDA receptors blockade controls the VTA’s DA mechanism for opiate reward processing (Tan et al., 2014). Therefore, the mPFC’s subregions were likely involved in the rewarding process of morphine.

Alternatively, the NAc and the BLA may modulate morphine reward and aversive conditioned learning (Bassareo et al., 2007; Bishop et al., 2011; De Luca et al., 2011; Yu et al., 2012). In one study, morphine-treated sensitization potentiated dopamine transmissions in the NAc core but not to the NAc shell and could therefore link to morphine-induced reward and aversion (De Luca et al., 2011). Significant increases have been noted in the kappa-receptor mRNA expression of the mPFC and its downstream NAc and VTA after acute morphine treatment. However, chronic morphine treatment did not show the same upregulation (Yu et al., 2012). Disruptions of the NMDA receptor of the PrL could enhance sensitivity to the rewarding property of the opiate, and this dysfunction was through the dopaminergic transmission in the BLA (Bishop et al., 2011). Therefore, the NAc and BLA may contribute to the rewarding and aversive conditioned learning by morphine.

The present data partially support this previous evidence. For example, our data showed that the Cg1 and PrL (but not the IL) of the mPFC, NAc core, NAc shell, and BLA demonstrated more c-Fos (%) after morphine-induced CTA for the 20, 30, or 40 mg/kg groups. Alternatively, the NAc shell did not show significant differences in c-Fos (%) in the CPP test in the 20–40 mg/kg groups. These results suggest that the different doses of morphine affect other brain areas [including the mPFC subareas (Cg1, PrL, and IL), NAc (NAc shell and NAc core), and the BLA] differently in terms of aversive and rewarding conditioned learning.

### 4.3 Expanding the paradoxical effect hypothesis of drug abuse: Neural association and contributions under various doses of morphine

The Cg1, PrL, IL, BLA, NAc, and DG showed more c-Fos expression after the morphine-induced CTA and CPP at a lower dose of 10 mg/kg of morphine (Yu et al., 2021). Moreover, our lesion study indicated that destroying the VTA with a high concentration of NMDA impaired morphine-induced CTA and CPP (Wu et al., 2021). A periaqueductal gray matter lesion did not change morphine’s aversive and reward conditioning at a 20 mg/kg dose of morphine (Wu et al., 2021). In conclusion, we suggested that the Cg1, PrL, IL, BLA, NAc, DG, and VTA were involved in the morphine’s CTA and CPP. These data supported the paradoxical effect hypothesis of abused drugs. However, the previous investigation of the paradoxical hypothesis did not examine different morphine doses or how different neural substrates were associated and involved in morphine’s aversion and reward in conditioned learning.

This study used varying concentrations of 20–40 mg/kg morphine injections in CTA and CPP tests. It showed that the positive association of the PrL and IL with the mPFC changed from the NAc core to the NAc shell as morphine doses increased from low to high in the CTA test. In the 20 mg/kg morphine group, the PrL was positively correlated with the NAc core and NAc shell; when the morphine dose was increased to 40 mg/kg, the positive correlation of the IL was changed with the BLA and NAc shell for the CPP test. Alternatively, the Cg1 and PrL appeared more c-Fos (%) in the 20–40 mg/kg groups; the NAc core, NAc shell, and BLA were more c-Fos (%) in the 30–40 mg/kg groups following CTA tests. In the reward CPP tests, the Cg1, PrL, IL, and BLA expressed more c-Fos (%) for the 20 mg/kg group; the c-Fos (%) of the Cg1 revealed more in the 40 mg/kg group; however, the c-Fos (%) of the NAc core was lower in 30–40 mg/kg morphine groups compared with the saline group. In conclusion, the present data expand the paradoxical effect hypothesis. The different morphine doses activated changes in substrate association, and the mPFC subregions (e.g., Cg1, PrL, and IL), NAc subareas (e.g., NAc core and NAc shell), and BLA were involved in the different doses of morphine injections after CTA and CPP tests.

### 4.4 Clinical implications

The evidence of the simultaneous existence of reward and aversion created by abused drugs challenges the conventional viewpoint of drug abuse solely being the result of drugs’ rewarding properties (Wang et al., 2010; Wu et al., 2021; Yu et al., 2021). The reward hypothesis of abused drugs proposes that the acute administration of abused drugs induces impulsive behaviors and that chronic drug administration elicits compulsive behaviors. In this model, misusers continuously experience the addictive cycle of preoccupation, anticipation, binge and intoxication, and withdrawal negative effect (Koob, 2017; Volkow et al., 2019). A recent review reported that the aversive effect of the abused drug was as crucial as the rewarding property in influencing the development of drug addiction (Riley et al., 2022). Some research has claimed that the aversive properties of abused drugs could be used for anti-addiction approaches by developing them to block or interfere with rewarding properties (He et al., 2019; Yu et al., 2021; Riley et al., 2022). A similar concept was previously employed for alcohol and nicotine addiction (Mann, 2004; Casarrubea et al., 2015; Berrios-Carcamo et al., 2022). For example, the anti-alcohol consumption drug disulfiram disinhibited acetaldehyde hydrogenase. This caused higher amounts of acetaldehyde levels in the plasma, resulting in flushing, nausea, headache, and increases in heart rate aimed at deterring alcohol consumption (Mann, 2004). Morphine drinking produced an aversively bitter taste, enhancing avoidance behavior and counteracting the rewarding property of morphine-induced approaching behaviors (Berrios-Carcamo et al., 2022). Acute nicotine administrations elicited aversive anxiety behaviors to decrease the probability of nicotine addiction (Casarrubea et al., 2015). These previous studies suggest that the present findings on aversion and reward caused by morphine might provide some implications and insights for clinical treatment. How exactly the aversive property interfered with the rewarding property to ameliorate drug abuse should be investigated further.

### 4.5 Experimental limitations

This study employed various doses of morphine to elicit CPP. However, before the rats experienced the CPP test, they also received the experimental procedure of the CTA test. Thus, we found that c-Fos (%) of the NAc core was significantly decreased compared with the saline group following the CPP test; c-Fos (%) was not different in the NAc shell for all doses of the morphine groups after the CPP test. The present data of the NAc core and NAc shell conflict with previous studies (Cacciapaglia et al., 2012; Cheng et al., 2020). The results following the CPP task might have been confounded by a prior CTA test and thereby led to the decrease c-Fos in the NAc core and NAc shell. The results were interesting. Morphine-induced aversive effect attenuated the rewarding effect of morphine, and this inhibitory effect was shown in the NAc shell and NAc core, which mediated reward and reinforcement. Despite the decrease in neuronal activity level, the morphine-induced rewarding effect is evident. These conflicting data between the behavioral and neural levels should be investigated further. Whether the reduction of morphine-induced CPP leads to developed a novel intervention also needs to be examined further.

In summary, further studies should examine this issue that whether the conditioned aversive learning inhibits the subsequent reward learning and the neural activity of the NAc core or NAc shell.

## Data Availability

The original contributions presented in the study are included in the article/Supplementary Material, further inquiries can be directed to the corresponding authors.

## Abbreviations

ANOVA, analysis of variance; BLA, basolateral amygdala; Cg1, cingulate cortex 1; CPA, conditioned place aversion; CPP, conditioned place preference; CTA, conditioned taste aversion; DG, dentate gyrus; IL, infralimbic cortex; mPFC, medial prefrontal cortex; NAc core, nucleus accumbens core; NAc shell, nucleus accumbens shell; PBS, phosphate-buffered saline; PBST, phosphate-buffered saline tween-20; PrL, prelimbic cortex; VTA, ventral tegmental area.
