# Supplementary material for: Neuronal activity of the medial prefrontal cortex, nucleus accumbens, and basolateral amygdala in conditioned taste aversion and conditioned place preference induced by different doses of morphine administrations in rats
Source: Front Pharmacol. 2023 Jan 24;14:1062169. doi: 10.3389/fphar.2023.1062169 (PMC9902353; doi:10.3389/fphar.2023.1062169)
Supplement: Supplementary file 1 [file DataSheet1.docx]

Supplementary Material

# Supplementary Data

All experimental data are uploaded to the website below.

https://www.dropbox.com/sh/a6fmtk3suom236w/AABMHZctDlgfubprNdnNSBova?dl=0

# Supplementary Figures and Tables

## Supplementary Figures


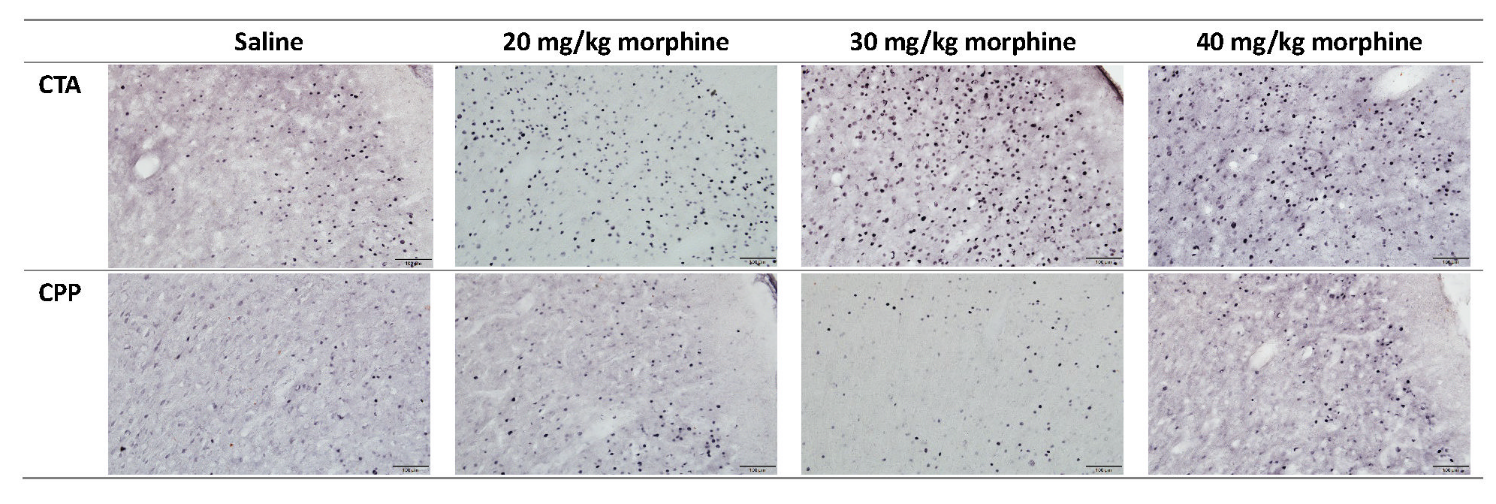
**Supplementary Figure S1.** Representative photomicrographs of c-Fos immunoreactivity for the Cg1 in the saline, 20 mg/kg, 30 mg/kg, and 40 mg/kg morphine groups after morphine-induced CTA and CPP conditioning. The scale bar represents 100 µm. Note: Cg1: cingulate cortex; CTA: conditioned taste aversion; CPP: conditioned place preference.


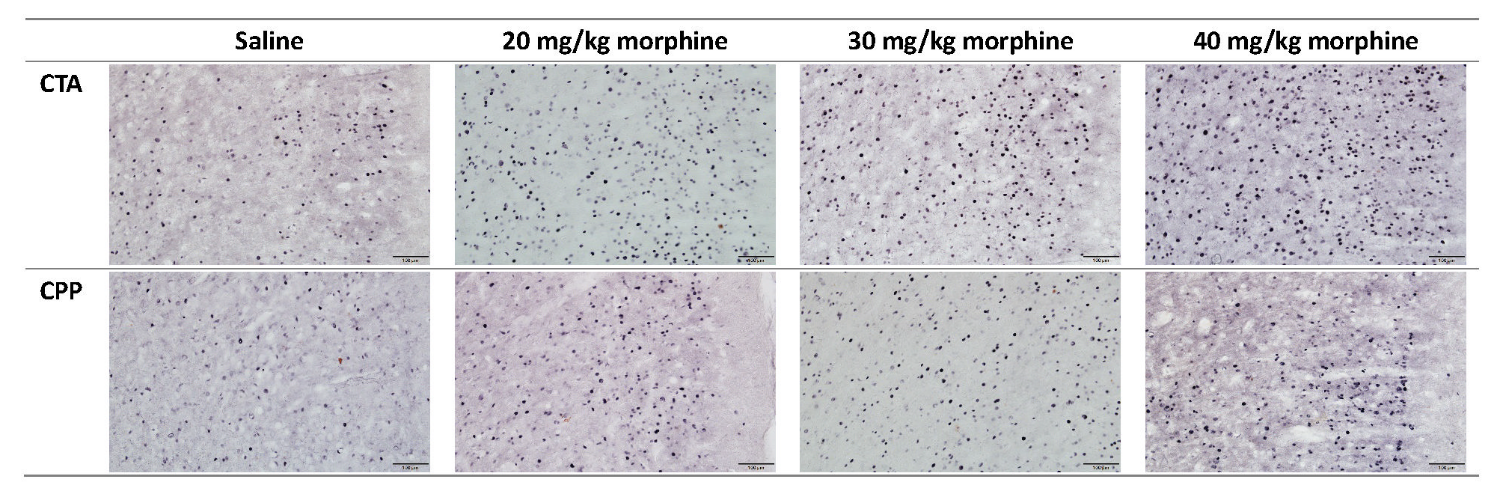
**Supplementary Figure S2.** Representative photomicrographs of c-Fos immunoreactivity for the PrL in the saline, 20 mg/kg, 30 mg/kg, and 40 mg/kg morphine groups after morphine-induced CTA and CPP conditioning. The scale bar represents 100 µm. Note: PrL: prelimbic cortex; CTA: conditioned taste aversion; CPP: conditioned place preference.


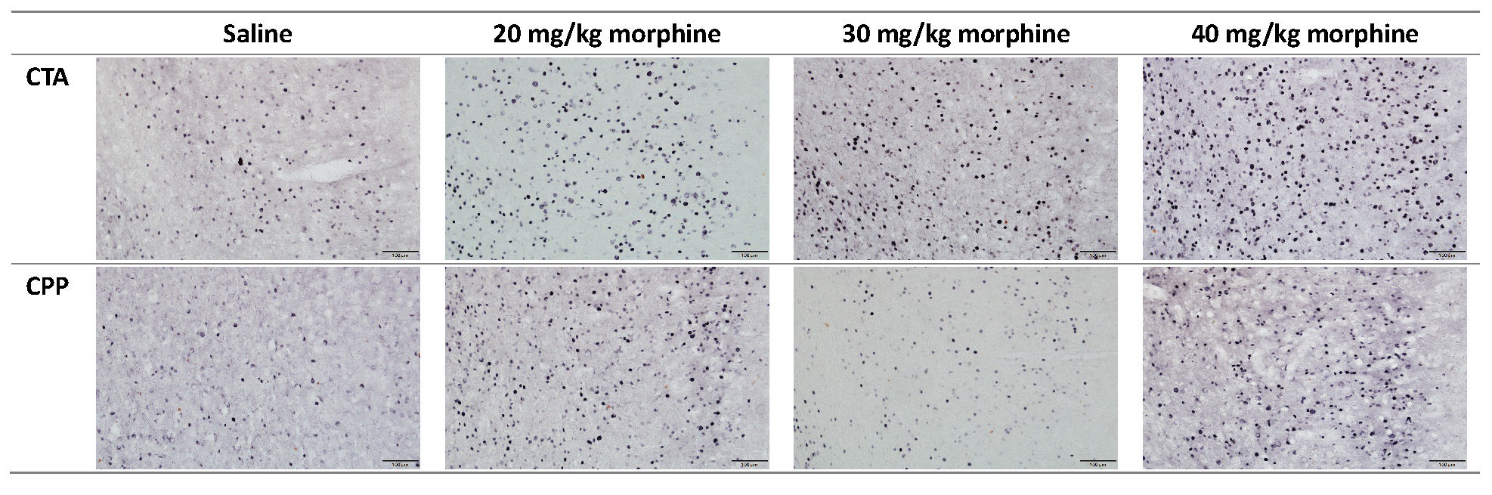
**Supplementary Figure S3.** Representative photomicrographs of c-Fos immunoreactivity for the IL in the saline, 20 mg/kg, 30 mg/kg, and 40 mg/kg morphine groups after morphine-induced CTA and CPP conditioning. The scale bar represents 100 µm. Note: IL: infralimbic cortex; CTA: conditioned taste aversion; CPP: conditioned place preference.


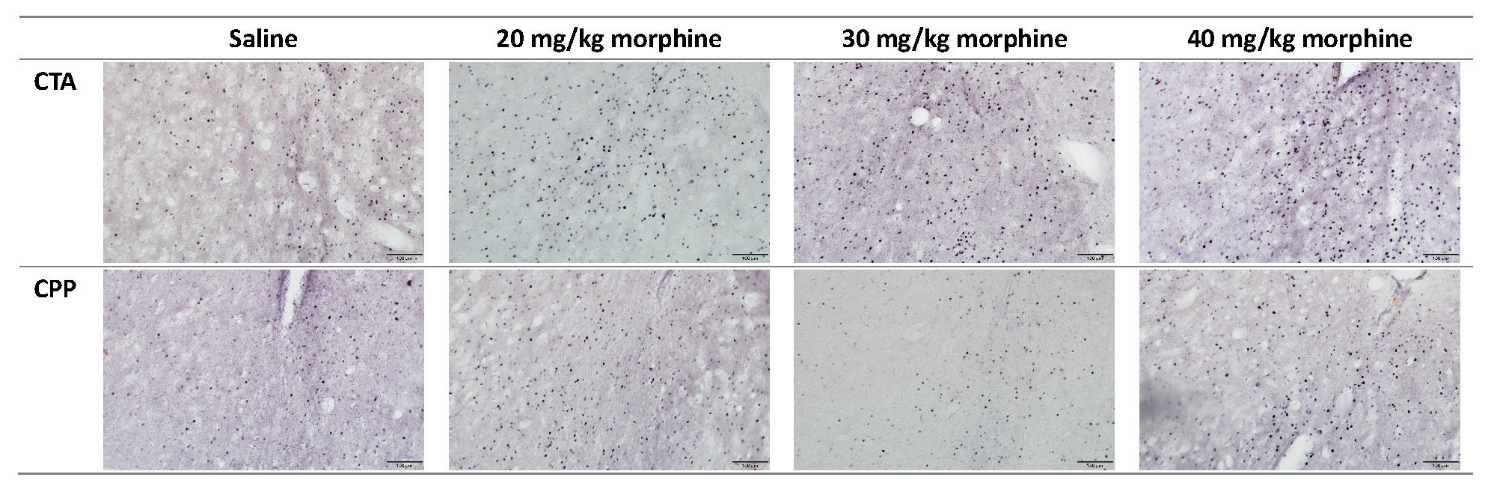
**Supplementary Figure S4.** Representative photomicrographs of c-Fos immunoreactivity for the NAc core in saline, 20 mg/kg, 30 mg/kg, and 40 mg/kg morphine groups after morphine-induced CTA and CPP conditioning. The scale bar represents 100 µm. Note: CTA: conditioned taste aversion; CPP: conditioned place preference.


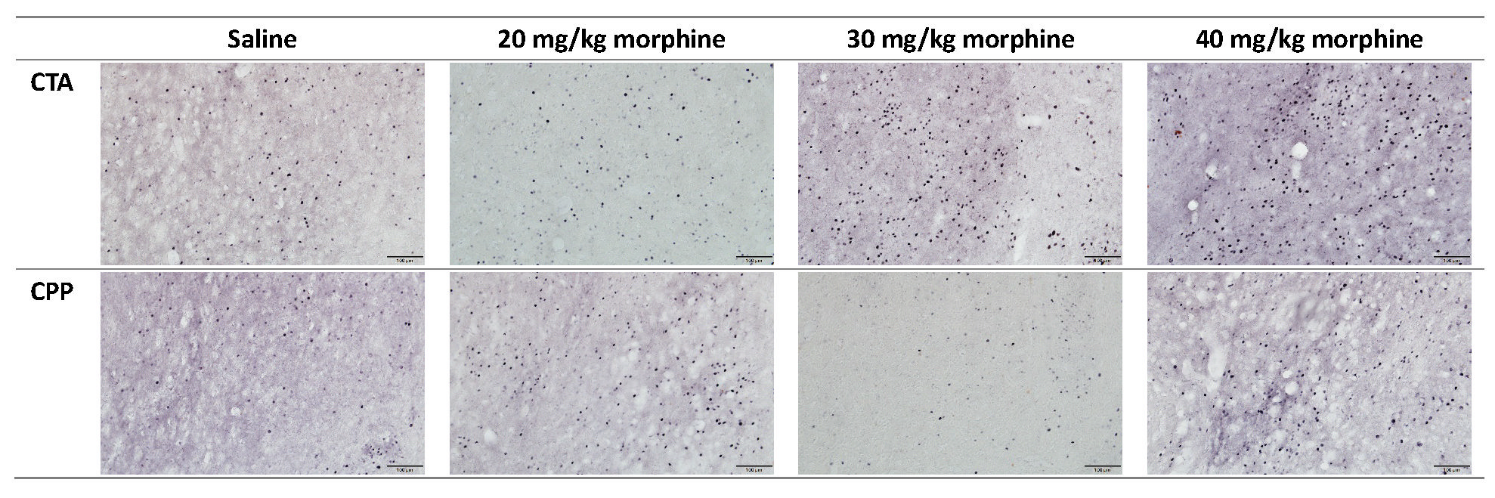
**Supplementary Figure S5.** Representative photomicrographs of c-Fos immunoreactivity for the NAc shell in saline, 20 mg/kg, 30 mg/kg, and 40 mg/kg morphine groups after morphine-induced CTA and CPP conditioning. The scale bar represents 100 µm. Note: CTA: conditioned taste aversion; CPP: conditioned place preference.


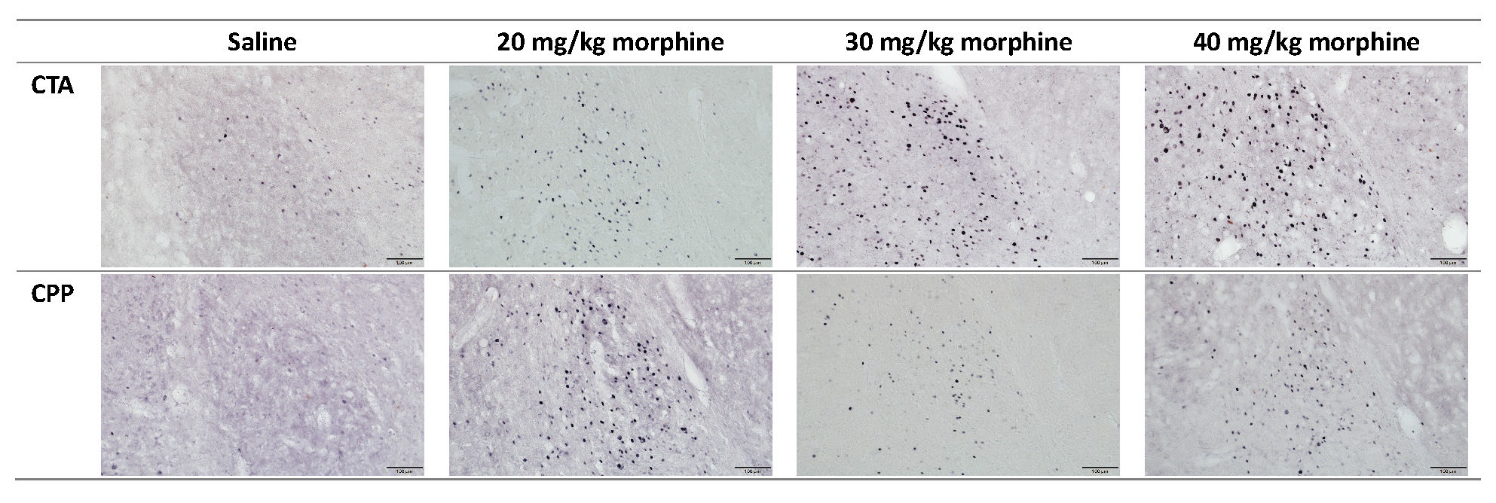
**Supplementary Figure S6.** Representative photomicrographs of c-Fos immunoreactivity for the BLA in the saline, 20 mg/kg, 30 mg/kg, and 40 mg/kg morphine groups after morphine-induced CTA and CPP conditioning. The scale bar represents 100 µm. Note: BLA: basolateral amygdala; CTA: conditioned taste aversion; CPP: conditioned place preference.
